# A Comparative Evaluation of the Effects of Three Remineralizing Agents on Bleached Enamel: A Scanning Electron Microscopy (SEM) Analysis

**DOI:** 10.7759/cureus.37240

**Published:** 2023-04-07

**Authors:** Neha Singh, Dipshi Ranjan, Shazia Mahreen, Ankita Pattanaik, Gurinder Kaur, Ajay Nagpal

**Affiliations:** 1 Department of Conservative Dentistry and Endodontics, Hazaribag College of Dental Sciences and Hospital, Hazaribagh, IND; 2 Department of Conservative Dentistry and Endodontics, Kanti Devi (KD) Dental College and Hospital, Mathura, IND; 3 Department of Oral and Maxillofacial Pathology, Kanti Devi (KD) Dental College and Hospital, Mathura, IND

**Keywords:** elsenz, tooth min, tooth mousse+, remineralizing agents, bleached enamel, scanning electron microscope

## Abstract

With the emerging concern of patients on esthetics, bleaching has become quite popular to get that "Shining White Smile." However, bleaching chemicals used on enamel's surface have been a clinical issue due to the fact that they might cause a variety of side effects, including sensitivity, gingival irritation, dentinal sensitivity, demineralization, and changes in the enamel's surface morphology. As a result, it is important to investigate different remineralizing agents that help to reduce the adverse effects.

The researchers in this in-vitro study used a scanning electron microscope (quanta 200 SEM, California, USA) and a universal testing machine to assess the effects of three remineralizing products such as Tooth Mousse Plus (GC Corporation, India), Tooth Min (Abbott, India), and Elsenz (Group Pharmaceuticals Ltd, India) on bleached enamel. Based on the data available, we determined that bleaching greatly reduces the enamel microhardness of permanent human premolars; however, this loss could be recovered with a remineralizing agent. Tooth Mousse Plus is the most effective remineralizing agent among the three, followed by Elsenz and, finally, Tooth Min.

## Introduction

Esthetics is one of the most important aspects of debilitating human personality. Patients seek cosmetic dentistry care for the elimination of any and all perceptions of discoloration in their front teeth. There are several potential reasons for tooth discoloration, which is defined as any alteration in the color, tint, or translucency of a tooth. Discoloration can be the result of several factors. Several modalities include laminates, crowns, and several teeth whitening methods [1]. To lighten discolored teeth, bleaching is the most popular method since it is quick, easy, and inexpensive [2].

Hydrogen peroxide or its precursor, carbamide peroxide, is the active component in the in-office and at-home bleaching gels in concentrations ranging from 3% to 40% of hydrogen peroxide equivalent. An oxidizing agent called hydrogen peroxide diffuses into teeth and breaks down into unstable free radicals. These radicals attack organic pigmented molecules that are sandwiched between inorganic salts in tooth enamel by attacking the double bonds of chromophore molecules in tooth tissues. The shift in the chromophore molecules' absorption spectrum and smaller, less pigmented constituents brought about by the change in double-bond conjugation causes the bleaching of tooth tissues [3]. However, it has been observed that bleaching agents have several negative effects on teeth and the surrounding structures, such as tooth sensitivity, gingival irritation, dentinal sensitivity, enamel demineralization, and surface morphological alterations [3]. Bleaching affects enamel primarily in three aspects: loss of minerals, changes in surface morphology, and alteration of surface microhardness. Despite these disadvantages, patients insist on bleaching treatment. As a result, it is important to investigate potential treatments, such as the use of remineralizing agents, that might reduce the frequency with which these adverse effects occur. To drive ion deposition into crystal gaps in demineralized enamel and produce net mineral gain, a tooth might undergo a process called remineralization, in which calcium and phosphate ions are given to the tooth from an external source [4].

Fluoride has been an agent of choice for remineralizing teeth for the last 60 years. This therapy leads to the formation of a calcium fluoride-like coating on the surface of enamel, protecting it from further acid assault and minimizing the mineral loss that follows it [5,6]. Sugar substitutes, ozone, fluorides, calcium sucrose phosphate, inorganic calcium phosphate, calcium phosphate remineralization, and casein phosphopeptide amorphous calcium phosphate fluoride (CPP-ACPF) are only a few of the remineralizing agents available. Bioactive glass with fluoride added has also been gaining popularity as a remineralizing agent in recent years [7].

For the remineralization of early enamel caries lesions, various technologies are employed. Three different technologies are used in this study. The technology behind ToothMin tooth cream is from anticay. Anticay is a substance that, by weight, has a calcium content between 10% and 12% and a phosphorous content between 8% and 10%. Elsenz is a unique toothpaste with fluoride, a bioactive glass containing fluoro-calcium phosphosilicate. The active ingredient in Tooth Mousse Plus is CPP-ACPF with 900 ppm fluoride. As calcium and phosphate compounds are essential to all systems, their effect is primarily based on an improvement in saliva's inherent ability to replenish the mineral loss. Clinical practitioners have given remineralization a lot of consideration as a therapeutic strategy.

Due to a lack of comparative studies between Tooth Mousse Plus, Elsenz, and ToothMin for their remineralization capabilities, the rationale of this in-vitro study is to evaluate and compare the efficacy of various remineralizing agents, such as Tooth Mousse Plus, ToothMin, and Elsenz, on bleached enamel using a scanning electron microscope (SEM) and a universal testing machine.

## Materials and methods

Fifty human premolars were sanitized after being removed for periodontal and orthodontic treatment and then examined for signs of caries, cracking, hypoplasia, and white spot lesions. The ethical clearance was obtained with IRB number: IEC/2020/122. After sterilization in a 5.25% sodium hypochlorite solution for one hour, they were maintained in artificial saliva for the duration of the experiment. A double-sided diamond disc was used to vertically segment the teeth into 100 enamel samples, which were then embedded in acrylic resin. These one hundred samples were split into five sets of 20.

These 100 samples were randomly divided into five groups of 20 samples in each group. Group 1 was the positive control group (no bleaching), and Group 2 was the negative control group (only bleaching). Group 3 (bleaching followed by application of Tooth Mousse+), Group 4 (bleaching followed by application of Elsenz), and Group 5 (bleaching followed by application of ToothMin) were experimental groups.

The teeth in Groups 2, 3, 4, and 5 were whitened using the teeth whitening equipment. The third, fourth, and fifth groups were treated with remineralizing agents such as Tooth Mousse+ (GC Corporation, India), Elsenz (Group Pharmaceuticals Ltd., India), and ToothMin (Abbott, India), respectively, for 15 minutes as this time is required for the remineralization to take place, after which they were rinsed off with distilled water. All of the samples were tested for surface hardness using a Vickers hardness tester (Reichert, Austria) by indenting the enamel surface with a 100-gram weight and observing how long it took for the indentation to settle and rest on the enamel surface (30 seconds). Scanning electron microscopy (quanta 200 SEM, California) was used to examine the variations on the surfaces of different groups. Gold-sputtered specimens were used. All teeth were picked based on the inclusion criteria and then randomized into groups. For objectivity, one dentist independently graded the roughness or smoothness of an enamel surface using SEM. The surface smoothness of enamel seems to have been significantly altered following bleaching as seen by the appearance of imperfections and holes detected by the SEM analysis.

The enamel surface changes (surface roughness) were examined and scored by one dentist as 0 - enamel with smooth surface morphology, 1 - enamel with slight irregularities, 2 - enamel with moderate irregularities, and 3 - enamel with accentuated irregularities.

Statistical analysis was used to decipher the differences and the importance between the groups in the current study's findings. The statistical analysis for the present study was conducted using the paired t-test and the Wilcoxon signed-rank test. Information was analyzed using the Statistical Package for the Social Sciences (SPSS) software, version 17.0 (SPSS Inc., Chicago) for Windows, a statistical program designed for social science research. The level of statistical significance was set at 95% (P = 0.05).

## Results

The result of our study was evaluated in terms of variation in microhardness and surface smoothness.

Vickers analysis for microhardness

The mean and standard deviation of the microhardness of all groups are given in Table 1.

**Table 1:** Mean and standard deviation of the microhardness of all groups SD: Standard deviation; N: Number; Min: Minimum; Max: Maximum.

Group	N	Mean	SD	Min.	Max.
Control group	10	294.30	10.51	277.00	310.00
After bleaching	10	245.90	10.46	231.00	262.00
Bleaching + Tooth Mousse Plus	10	277.80	11.31	255.00	293.00
Elsenz	10	271.60	8.22	262.00	284.00
ToothMin	10	266.90	13.72	249.00	287.00

The results of the comparison of microhardness between various groups are given in Table 2.

**Table 2:** Paired t-test for comparison between various groups S: Significant; NS: Not significant; SD: Standard deviation.

	Paired t-test	N	Mean	SD	Paired mean differences	t-test	P-value	Inferences
Pair 1	Before bleaching (control)	10	294.30	10.51	48.40	10.116	0.001	S
	After bleaching	10	245.90	10.46				
Pair 2	Before bleaching (control)	10	294.30	10.51	16.50	3.787	0.004	S
	Bleaching + Tooth Mousse Plus	10	277.80	11.31				
Pair 3	Before bleaching (control)	10	294.30	10.51	22.70	5.404	0.001	S
	Bleaching + Elsenz	10	271.60	8.22				
Pair 4	Before bleaching (control)	10	294.30	10.51	27.40	5.629	0.001	S
	Bleaching + tooth min	10	266.90	13.72				
Pair 5	After bleaching	10	245.90	10.46	-31.90	18.881	0.001	S
	Bleaching + Tooth Mousse Plus	10	277.80	11.31				
Pair 6	After bleaching	10	245.90	10.46	-25.70	10.433	0.001	S
	Bleaching + Elsenz	10	271.60	8.22				
Pair 7	After bleaching	10	245.90	10.46	-21.00	4.131	0.003	S
	Bleaching + ToothMin	10	266.90	13.72				
Pair 8	Bleaching + Tooth Mousse Plus	10	277.80	11.31	6.20	2.418	0.039	S
	Bleaching + Elsenz	10	271.60	8.22				
Pair 9	Bleaching + Tooth Mousse Plus	10	277.80	11.31	10.90	2.386	0.041	S
	Bleaching + ToothMin	10	266.90	13.72				
Pair 10	Bleaching + Elsenz	10	271.60	8.22	4.70	0.914	0.385	NS
	Bleaching + ToothMin	10	266.90	13.72				

The results of our study suggest that there is a significant decrease in microhardness after the bleaching procedure. All experimental groups showed an increase in microhardness after the application of remineralizing agent on bleached enamel, although it is significantly lesser than the unbleached enamel. Among all three remineralizing agents, Tooth Mousse Plus showed the maximum increase in microhardness, followed by Elsenz and, least with, ToothMin. However, Elsenz and ToothMin showed no significant difference in remineralizing capability: Group 1 > Group 3 > Group 4 > Group 5 > Group 2.

The results of our study suggest that there is a significant increase in surface roughness after the bleaching procedure. The application of remineralizing agent after bleaching definitely improves the surface roughness and results in a smoother surface. These enhancements were significant in cases of Tooth Mousse Plus and Elsenz but not significant in cases of ToothMin: Group 1 > Group 3 > Group 4 > Group 5 > Group 2.

## Discussion

It is impossible to ignore the bleaching chemicals' negative effects on oral tissues. Bleaching chemicals cause a decrease in enamel microhardness due to their oxidation and reduction processes, which disintegrate organic and inorganic tooth components (demineralization) [8]. While amorphous calcium phosphate (ACP) technology had been around since the early 1990s, it was not until 1998 that CPP-ACP was used as a remineralizing agent [9]. Tooth Mousse Plus contains recaldent, a milk-based protein that replenishes the minerals that wear away at the enamel of teeth. CPPs inhibit demineralization by stabilizing high levels of ACP on the tooth surface, which in turn promotes the remineralization of enamel [10]. CPP-ACPF with 900 ppm fluoride is the active component in Tooth Mousse Plus. The results of our research showed that the Tooth Mousse Plus product provided much more protection than the original Tooth Mousse [1]. Fluoride has a synergistic effect on the remineralization of early carious lesions when combined with CPP-ACP. The ongoing processes of re-precipitation and dissolution at the tooth-oral fluid interface are changed by the fluoride present in the oral fluids. Incipient caries lesions' remineralization is sped up by traces of fluoride [1].

Elsenz, a bioactive glass containing particles of fluoro-calcium phosphosilicate, is a brand-new product. Elsenz gradually releases calcium, phosphate, and fluoride ions over the course of 8-12 hours to produce the fluorapatite mineral, which is used to restore, strengthen, and protect tooth structure [11]. The exodus of hydroxycarbonate apatite (HCA) is a potential mode of action of bioactive glass. According to Arnold et al., when these particles cling to teeth, they produce a coating of HCA, which is thought to aid in the remineralization process [11]. Anticay technology is the foundation of ToothMin tooth cream. Anticay is a compound that contains 10%-12% of calcium and 8%-10% of phosphorous by weight. Both calcium sucrose phosphates and inorganic calcium phosphates are the components. While being broken down into individual forms, calcium and phosphorus release carbon dioxide. Enamel remineralization speeds up when calcium and phosphate ions bind to its surface, decreasing its solubility [12]. Plaque development is also reduced as a result of this. The large amounts of free calcium and phosphate ions it provides are many times higher than those naturally present in saliva, which explains why it is so powerful at remineralizing teeth [13].

We undertook this research because no studies had previously compared the remineralization abilities of Tooth Mousse Plus, Elsenz, and ToothMin. The Vickers hardness test was used to calculate the microhardness of the teeth's surfaces because it is a simple method for evaluating the mineral loss or gains inside the teeth's structure and, hence, its mechanical characteristics. Micro-indentation testing is often used to identify the changes in the enamel and dentin surfaces after de-mineralization and remineralization as it is a nondestructive means of analyzing the mineral changes that have occurred due to therapeutic therapies. The procedure is straightforward, rapid, and straightforward to measure, allowing for repeated measurements of the same specimen over a given time period, which in turn reduces experimental variance. Our research suggests that surface hardness decreases as a consequence of bleaching. Tooth Mousse Plus was the most effective remineralizing product, followed by Elsenz and finally by ToothMin. All three groups had an increase in surface microhardness after using remineralizing agents.

The findings of Soares et al. were proven by these analyses. The self-assembling peptide P11-4 was shown to be the most successful at remineralizing enamel out of the four different materials examined (CPP-ACPF, bioactive glass [BAG], fluoride-enhanced hydroxyapatite [HA] gel, and P11-4) [14].

These results are consistent with those found by Klarić et al., who studied the effects of two in-office bleaching agents on human enamel and dentin and found that the bleaching procedure significantly alters the smoothness of the enamel and dentin surfaces, resulting in slight irregularities [15]. More so, the study of the surface morphology of enamel after the application of 35% hydrogen peroxide by Poggio et al. revealed considerable surface alterations and abnormalities [16].

Group 3 patients who used Tooth Mousse Plus after bleaching their teeth had the greatest improvement in enamel remineralization and protective layer deposition. Our findings were consistent with those found by Poggio et al. After conducting experiments on dyed finish surfaces with various remineralizing specialists (Remin Pro, Tooth Mousse, MI Glue, and Profluorid varnish), they discovered that CPP-ACP (MI in addition to glue) formed a total and homogeneous defensive layer, making it more effective at remineralization [16].

Some demineralization evidence (pores and irregularities) was still visible on the surface after applying Elsenz and ToothMin to the bleached enamel surface in groups 4 and 5. Inconsistencies in the mechanical characteristics of enamel, changes in tooth structure with age, medication effects, absorbed fluoride content, orientation and density of hydroxyapatite crystals, moisture of specimens, and research technique were the major drawbacks of this experimental setup. Exposure to bleaching chemicals may cause demineralization of enamel, which can be affected by all of these causes [17]. Karlinsey et al. [18] discovered that remineralizing bovine enamel samples with CPP-ACP + fluoride was successful. In the present research also, CPP-ACP + fluoride is found to be more effective than other agents. The oral pH fluctuates often based on a person's diet and oral hygiene routine. So, it is hard to recreate the mouth's conditions accurately [11]. An extraoral study's findings may vary according to a number of factors, including the pH of bleaching chemicals and how the specimens were stored [9]. The small sample size was one of the study's limitations. A higher number of samples is recommended for future studies.

## Conclusions

Within the limitation of our study, it can be concluded that the enamel microhardness of permanent human premolars was significantly decreased following bleaching. The application of remineralizing agents increases microhardness. Among the three, Tooth Mousse Plus is most effective as remineralizing agent followed by Elsenz and, least by, ToothMin.
